# Association of filial attitude, filial behavior and death literacy: implications for development of death system in Guangdong-Hong Kong-Macao Greater Bay Area of China

**DOI:** 10.1186/s12889-024-18197-3

**Published:** 2024-03-06

**Authors:** Wai I Ng, Sok Leng Che, Xiang Li, Mingxia Zhu

**Affiliations:** 1 Education Department, Kiang Wu Nursing College of Macau, Macao SAR, China; 2 Nursing and Health Education Research Centre, Kiang Wu Nursing College of Macau, Macao SAR, China

**Keywords:** Filial attitudes, Filial behaviors, Death literacy, Adult children, Dying parents

## Abstract

**Background:**

Filial piety, as a major traditional norm in Chinese culture and in Chinese families, affects the attitudes and behaviors of adult children toward their parents and impacts their end-of-life decision-making and the quality of death of their parents. Death literacy is a novel concept aimed at promoting palliative care in the context of public health.

**Aims:**

To understand attitudes and behaviors related to filial piety and to examine the role of death literacy in filial behaviors toward dying parents among residents in the Guangdong-Hong Kong-Macao Greater Bay Area of China.

**Methods:**

A cross-sectional online survey that employed the convenient and snowball sampling methods was adopted. Filial Piety Representations at Parents’ End of Life Scale and Death Literacy Index were used.

**Results:**

This study identified a significant gap between the filial piety attitudes and behaviors of Chinese adult children. Gender, caregiving experience and death literacy were predictors of filial behaviors in an end-of-life context.

**Conclusion:**

Providing truth disclosure support, offering guidance to young adult children and caregivers of terminally ill fathers, and strengthening factual and community knowledge of death are necessary to enhance the reciprocal comfort of both adult children and dying parents in the context of Chinese filiality.

## Introduction

Filial piety is the core sociocultural value featuring the Chinese society for thousands of years, which emphasizes moral obligation in a strict hierarchical sense within an extended family based on the recognition of aid and care given by the seniors. Comprehensive integration of such traditional value into decision-making of personal issues at any stages of life, ranging from the planning of career path and marriage to even the way of end-of-life (EoL) care [1–6], is considered as an important factor for shaping a “proper” interaction pattern among family members to maintain familial and social stability. Thus far, the attitudes and behaviors of individuals from hundreds of millions of households residing in Greater China, including Hong Kong and Macau, are still significantly constrained by the norms constituting the idea of filial piety. Individuals fail to adhere to the obligation may be undergone moral sanctions, such as social exclusion. As such, individuals originated from typical Chinese families generally use a collective and family-centered approach as the basis for decision-making [7], which may end up with the sacrifice of true personal needs or feelings. In fact, it has been found that the implanted idea of filial piety in adult children is associated with the level of parental well-being, including higher life satisfaction and lower mortality [4, 8–12].

Although the nowadays notion of a good death focusing on individualism as defined in Western medicine has been increasingly conceptualized into the context of familism in traditional Chinese philosophy [13], the topic of death still remains rarely mentioned by families. During the course of EoL care, Chinese adult children intentionally, or sometimes unconsciously, avoid to become the ill-omened bird flying over their terminally ill parents in order to fulfill the desire to uphold filial piety [3, 14]. One of the explanations for such behavior is the offensive nature of discussing death in Chinese society, which makes it a taboo or cursed subject [14]. On the other hand, to withhold bad news from parents close to the end of their life is considered as a social good to protect parents from the stress of death by promoting the sense of peace in their mind [15]. Therefore, if adult children induce the discussion of death with their parents or seniors, they tends to believe that they are or might be regarded as unfilial [3]. Under such circumstances, terminally ill parents may have died without being aware of their actual situation, leading eventually to the poor preparation for death. Since good quality of death has been widely recognized as the result of planned death [16], the practice of filial piety could compromise the pursuit of good death. Owing to the increasing interest in the quality of death among Chinese population in recent years, together with the findings which point towards the necessity of including filial piety in the promotion of good death [14, 15, 17], it is important to better understand the nature of filial piety and its association with decision-making of EoL caring.

Yeh and Bedford [11] developed a dual model to classify filial piety according to two practicing approaches namely the authoritarian and reciprocal approach which elucidates the representations of filial piety in everyday existence. In brief, authoritarian filial piety manifests as obedience and obligation of children to parent’s expectation, while the reciprocal approach manifests as interactive and affective relationship between children and parents. Several studies have reported that the behavior of parent-child communication and relationship is significantly different in families practicing the two different approaches of filial piety [18–21]. Of note, reciprocal filial piety has been found to have a positive association with EoL discourse as it encompasses intimacy and emotional caring in the parent-child relationship [7, 17]. In addition, such more interactive approach has been shown to supportively mediate the relationship between close adult attachment and caregiver preparedness [22], which may serve potentially as a factor to facilitate the quality of death among Chinese population thereof. In accordance with such findings and based on the dual model of filial piety, Che et al. [23] developed the Filial Piety Representations at Parents’ End of Life Scale (FPR-EoL) to specifically assess the influence of filial piety in the context of adult children confronting the dying process of their parents. The researchers identified four distinct factors (or four separated stages) in this process among adult children of the Chinese population, which are critically influenced by filial piety representations: (1) “Disclosing Bad News” involving the disclosure of prognosis to parents; (2) “Respect and Comfort” involving the preparations (even included self-sacrifice) that were done to comfort the dying parents by satisfying their wishes or expectations; (3) “Acceptance of Death” involving the acknowledgment of the truth of the parents’ dying without resentful about it; (4) “Spending Final days” involving the preparation of oneself and parents for death.

**Fig. 1:**
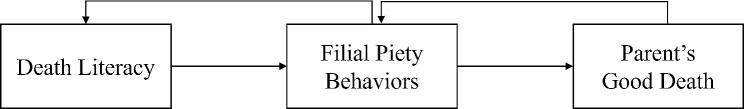
Conceptual Framework

Further investigation of Chinese filiality have suggested a decomposition of filial piety into filial attitudes and filial behavior [24]. Intriguingly, it has also been found that the attitudes of filial duties are not strongly associated with filial behavior [25]. Such discrepancies between one thought they should do and what they actually do may result in unpredictable outcome of the quality of one’s death in the context of EoL decision-making. Therefore, the exploration of such discrepancies may help inform filial behavior, and to clarify the role of filial piety in one’s death in an EoL context is of pressing need. Of note, Death literacy is a novel concept that was introduced in 2018 [26], which refers to the cognitive and practical ability of an individual to approach, understand and make informed choices about EoL and options pertaining one’s death. It has been claimed that people with good death literacy are aware of the death system in their community and can obtain access to the EoL care services. However, the examination of the associations between death literacy and other attributing factors for quality EoL is still limited. Recently, the experiences of caring for dying people and quality of death of parents has been found to associate with better level of death literacy [27, 28]. On the other hand, a higher death literacy has been suggested to enable one the individual ability to manage EoL and death issues [29]. Taking into account that EoL and death issues are the main concerns in filial representations at parent’s EoL among Chinese families [23], it is hypothesized that filial behavior to be associated with one’s death literacy in this study as illustrated in Fig. 1.

In the current study, we particularly focused on exploring the representations of filial piety in an EoL context exhibited by residents in the Guangdong-Hong Kong-Macao Greater Bay Area (GBA), based on the countrywide policy of developing this area into an integrated region for economic development and ideal living [30]. This study aims to examine residents’ discrepancies in attitudes and behaviors pertaining to filial piety, and to examine the role of death literacy in filial behavior toward parents who are at the end stage of their lives.

## Methods

This study featured a cross-sectional survey that was administered using convenience and snowball sampling methods. An online questionnaire was disseminated from October to November 2022 via email and social media, including Facebook, WhatsApp and WeChat, in five cities in the GBA, namely, Guangzhou, Jiangmen, Zhuhai, Hong Kong and Macao. Participants were residents (residing for 6 months or above) from the five cities above, aged between 18 and 74 years and were able to read Chinese were recruited. The sample size was calculated based on the suggestion of [31], requiring the sample size of at least 600.

An introduction to the study was included at the beginning of the online questionnaire. Participants were asked to read the information, to select ‘yes’ according to the three provided criteria as mentioned above, and to select the ‘consent’ option before starting the survey. The questionnaire consisted of three sections. The first section focused on participants’ backgrounds, including demographic data such as their age, gender, education level, religion, their living status with their parents, and their relationship with their parents. The second section included the Filial Piety Representations at Parents’ End of Life Scale (FPR-EoL), in which both the degree to which participants agreed with the items and their tendency to act were collected. The third section was the Death Literacy Index (DLI).

### Filial piety representations at parents’ end of life scale (FPR-EoL)

The FPR-EoL was developed and validated by Che et al. [23]. The FPR-EoL is a 19-item scale measures the filial piety representations of Chinese adult children when their parents are at the end of their lives. The scale consists of four subscales: (1) respect and comfort, (2) acceptance of death, (3) spending final days, and (4) disclosing bad news. It exhibited a reliability of Cronbach’s alpha of 0.73. This study examined the discrepancies between the participants’ attitudes and behaviors in terms of filial piety when they encountered parents’ EoL. In this context, the responses of the questionnaire were scored in two ways. Respondents could select an option on a scale ranging from ‘extremely agree’ (a score of 5) to ‘extremely disagree’ (a score of 1) with respect to their filial piety representations with the goal of assessing their attitudes with regard to filial piety in an EoL context; in contrast, filial piety-related behavior in an EoL context was measured by asking respondents to select an option on a scale ranging from ‘extremely tend to do’ (a score of 5) to ‘extremely not tend to do’ (a score of 1) in response to the given filial piety representations. Higher scores indicate greater levels of agreement with filial piety behaviors and a greater tendency to put them in practice.

### Death literacy index (DLI)

The DLI was developed and validated by Leonard and colleagues in 2020 as part of the Groundswell Project of Australia [29, 32]. This measure is used to assess the knowledge and skills that enable people to gain access to, to understand, and to make informed choices about death and end-of-life care options. This notion serves as an index that can be used to predict the death quality of individuals as well as to reflect the overall quality of public health palliative care [33]. DLI is a 29-item scale comprising 4 subscales: ‘Practical Knowledge’ (8 items), ‘Experiential Knowledge’ (5 items), ‘Factual Knowledge’ (7 items) and ‘Community Knowledge’ (9 items). Higher DLI scores indicate better death literacy [32]. The DLI has been translated into Swedish [34], Turkish [35] and Chinese [36]. This study utilized the Chinese version, which was validated by Che et al. [36] as the participants in this study were all Chinese. Its internal consistency was good, with an overall Cronbach’s alpha of 0.94.

### Statistical analysis

Descriptive analysis was used to demonstrate demographic data, representations of filial piety, and death literacy. Paired t tests were used to compare the FPR-EoL (attitude) and FPR-EoL (behavior) scores of the participants, and the difference between attitudes and behaviors pertaining to filial piety. As behavior is more closely associated with the actual actions taken by the individual, FPR-EoL (behavior) was subjected to further inferential statistical analysis, including a one-way ANOVA used to examine the differences in FPR-EoL (behavior) exhibited by participants with different demographic characteristics. Post hoc analysis was used to examine the differences among different age groups and occupations.

A multiple linear regression analysis with enter method was conducted to identify factors contributing to FPR-EoL, and to examine the role of death literacy in filial behavior toward parents who are at the end stage of their lives. Variables only significantly associated with FPR-EoL (behavior) in one-way ANOVA were entered in the model and were treated as independent variables to identify predictors. The threshold of the *p* value in this study was set to ≦ 0.05. There was no missing data in the collected data. The Statistical Package for the Social Sciences (SPSS) version 22 software was used to conduct the analysis mentioned above.

## Results

A total of 6,059 people from five cities of the GBA of China participated in the survey and 3052 questionnaires were valid. Invalid questionnaires included those who did not live in one of the five cities, those who expressed and their unwillingness to participate, those whose age did not fulfill the criteria, those whose parents had passed away, and those incompleted questionnaires. Females accounted for 79.5% (*n* = 2425) of the participants. Participants were aged between 18 and 74 years (28.3 ± 12.4). Most of the participants had received high school level of education or below (60.3%, *n* = 1840), were not married (70.7%, *n* = 2158), had no children (73.9%, *n* = 2254) but did have siblings (81.7%, *n* = 2459). Most of the participants had no religious beliefs (74.1%, *n* = 2263), with income below the city’s average (61.1%, *n* = 1865), were students, had not been hospitalized within the previous year (95.6%, *n* = 2917) and had two living parents (87.7%, *n* = 2677).

In addition, several participants’ characteristics were associated with differences in FPR-EoL (behavior) scores, including being female (*t*=-2.47, *p* = 0.014), being 35 years old or above (*F* = 55.95, *p* < 0.001), had reveived college level of education or above (*t*=-12.77, *p* < 0.001), being married/cohabiting (*t* = 9.07, *p* < 0.001), having children (*t*=-7.84, *p* < 0.001), with income above the city’s average (*t*=-11.52, *p* < 0.001), being a nonstudent (*F* = 55.42, *p* < 0.001) and had been hospitalized within the previous year (*t*=-2.54, *p* = 0.01), all of those reported significantly higher FPR-EoL scores (behavior). Regarding the status of the parents of the participants and the participants’ relationships with their parents, those who had experienced death of their father (*t*=-4.98, *p* < 0.001), those who did not live with their parents (both *p* < 0.001), those who were not financial supporters of their parents (Father *p* = 0.004; mother *p* = 0.01), those who did not provide care for their parents (both *p* < 0.001), those whose parents had diseases (both *p* < 0.001) and those whose parents had been hospitalized within the previous year (both *p* = 0.01) reported significantly higher scores with regard to filial behavior in n EoL context. No significant differences were found in other variables for scores pertaining to EoL filial behavior in an EoL context (Table 1).

**Table 1:** Comparison of the FPR-EoL (behavior) score of the characteristics of participants and their parents (*n* = 3052)

Variables		n	%	Mean	SD	F/ t	p-value
Gender						-2.47	0.014
	Male	627	20.5	68.04	6.15		
	Female	2425	79.5	68.71	6.06		
Age (year)						55.95^b^	< 0.001
1	18–34	2311	75.7	67.93	5.86		1 < 2; 1 < 3
2	35–54	679	22.2	70.60	6.22		
3	55–74	62	2.0	70.52	7.25		
Highest Level of Education						-12.77	< 0.001
	High school or below	1840	60.3	67.45	5.72		
	College of higher	1212	39.7	70.29	6.22		
Marital Status						9.07	< 0.001
	Married/ cohabiting	894	29.3	70.11	6.03		
	Single/ divorced/ separated/ widowed	2158	70.7	67.94	5.99		
Children						-7.84	< 0.001
	Yes	798	26.1	70.01	6.08		
	No	2254	73.9	68.07	6.00		
Siblings						-0.65	0.52
	Yes	2495	81.7	68.61	6.01		
	No	557	18.3	68.42	6.38		
Religion						-1.66	0.10
	Yes	789	25.9	68.89	6.34		
	None	2263	74.1	68.46	5.99		
Income						-11.52	< 0.001
	Below city average	1865	61.1	67.58	5.82		
	Above city average	1187	38.9	70.13	6.16		
Occupation						55.42^b^	< 0.001
1	Medical (assistant) professional	719	23.6	70.06	5.98		1 > 2; 2 < 3; 2 < 4
2	Student	1688	55.3	67.34	5.72		
3	Other	533	17.5	70.00	6.16		
4	Not working	112	3.7	70.88	7.32		
Hospitalized within the past year						-2.54	0.01
	Yes	135	4.4	69.87	5.88		
	No	2917	95.6	68.51	6.08		
Parents living status						-4.98	< 0.001
	both alive	2677	87.7	68.36	5.98		
	at least one died/ don’t know	375	12.3	70.14	6.58		
Survival status of father						-5.15	< 0.001
	Alive	2753	90.2	68.39	5.99		
	Died/ don’t know	299	9.8	70.29	6.61		
Father’s age (years) (*n* = 2753)						53.44^a^	< 0.001
1	< 65	2174	79.0	66.93	6.23		1 < 2; 1 < 3
2	65–74	400	14.5	70.07	6.44		
3	>=75	179	6.5	69.74	6.56		
Living with father (*n* = 2753)						7.04	< 0.001
	Yes	1381	50.2	67.59	5.91		
	No	1372	49.8	69.19	5.98		
Financial supporter of father (*n* = 2753)						2.88	0.004
	Yes	1720	62.5	68.13	5.89		
	No	1033	37.5	68.81	6.13		
Carer of father (*n* = 2753)						5.35	< 0.001
	Yes	1515	55.0	67.84	5.91		
	No	1238	45.0	69.06	6.03		
Closeness with father (*n* = 2753)						1.40	0.16
	Not close/ very not close/ fair	990	36.0	68.60	5.97		
	Close/ very close	1763	64.0	68.27	6.00		
Health status of father (*n* = 2753)						1.11	0.27
	Fair/ bad/ very bad	1017	36.9	68.55	5.82		
	Good/ very good	1736	63.1	68.29	6.10		
Father with disease (*n* = 2753)						-7.56	< 0.001
	Yes	740	26.9	69.80	5.90		
	No	2013	73.1	67.87	5.94		
Father hospitalized within the past year (*n* = 2753)						-2.50	0.01
	Yes	292	10.6	69.22	5.80		
	No	2461	89.4	68.29	6.01		
Survival status of mother						-1.66	0.10
	Alive	2964	97.1	68.54	6.07		
	Died/ don’t know	88	2.9	69.64	6.54		
Mother’s age (*n* = 2964)						52.36^a^	< 0.001
1	< 65	2367	79.9	67.11	6.35		1 < 2; 1 < 3
2	65–74	387	13.1	70.17	6.51		
3	>=75	210	7.1	69.96	6.62		
Living with mother (*n* = 2964)						6.65	< 0.001
	Yes	1523	51.4	67.83	5.98		
	No	1441	48.6	69.30	6.06		
Financial supporter of mother (*n* = 2964)						2.45	0.01
	Yes	1889	63.7	68.34	6.00		
	No	1075	36.3	68.91	6.17		
Carer of mother (*n* = 2964)						5.32	< 0.001
	Yes	1742	58.8	68.05	6.02		
	No	1222	41.2	69.25	6.06		
Closeness with mother (*n* = 2964)						-0.56	0.57
	Not close/ very not close/ fair	525	17.7	68.41	5.97		
	Close/ very close	2439	82.3	68.57	6.09		
Health status of mother (*n* = 2964)						3.76	< 0.001
	Fair/ bad/ very bad	1127	38.0	69.08	6.00		
	Good/ very good	1837	62.0	68.22	6.08		
Mother with disease (*n* = 2964)						-10.18	< 0.001
	Yes	824	27.8	70.34	6.19		
	No	2140	72.2	67.85	5.88		
Mother hospitalized within the past year (*n* = 2964)						-2.70	0.01
	Yes	287	9.7	69.46	5.90		
	No	2677	90.3	68.44	6.08		

a Homogeneity of variance was assumed, Scheffe method was used as post-hoc comparison

b Homogeneity of variance was not assumed, Games-Howell method was used as post-hoc comparison

SD standard deviation

Table 2 shows all four subscales and the overall score of the FPR-EoL. Statistically significant differences were found between attitude and behavior, with the behavior score being higher than the attitude score (*p* < 0.001) with the exception of the subscale of ‘disclosing bad news’.

**Table 2:** Differences between FPR-EoL attitude and behavior scores (*n* = 3052)

Subscales	Attitude	Behavior	difference	t	p
Respect and Comfort	31.87	32.20	-0.34	-8.15	< 0.001
Acceptance of Death	14.16	14.68	-0.51	-12.13	< 0.001
Spending final days	16.62	17.05	-0.43	-11.40	< 0.001
Disclosing bad news	5.10	4.65	0.46	17.68	< 0.001
Overall Score	67.75	68.57	-0.82	-9.99	< 0.001

The total mean DLI score was 6.74 (SD = 1.51), in which context the highest score was in experiential knowledge (7.45, SD = 1.80) and lowest score was in community knowledge (6.25, SD = 2.08) (Table 3).

**Table 3:** Mean scores of Death Literacy Index (*n* = 3052)

Items		Scaled Mean	Std. Deviation
Practical Knowledge (8 items)		7.26	1.57
	Talking support (4 items)	6.72	1.99
	Hands on support (4 items)	7.80	1.84
**Factual Knowledge (7 items)**		**6.25**	**2.08**
**Experiential Knowledge (5 items)**		**7.45**	**1.80**
**Community Knowledge (9 items)**		**6.26**	**2.27**
	Accessing other’s help (5 items)	6.29	2.37
	Community support group (4 items)	6.21	2.54
**Total (29 items)**		**6.74**	**1.51**

All scales ranged 0–10

According to the results of the multiple linear regression, the FPR-EoL (behavior) of the participants was associated with their gender, age, being parents, being supporters of their mothers, their mothers had diseases and four subscales of the DLI. Female participants reported FPR-EoL (behavior) scores that were 0.75 higher than those reported by male participants (*p* = 0.01). Participants aged between 35 and 54 reported scores that were 1.07 points higher than those reported by participants aged 18 and 34 (*p* = 0.01). Participants with children scored 1.27 points less than participants without children (*p* = 0.01). Regarding parent-related factors, participants who were financially supporters of their mothers scored 1.06 points higher than participants who were not financially supporters of their mothers (*p* = 0.03). Participants whose mother had diseases scored 1.12 points higher than those whose mother did not have diseases (*p* < 0.01). Among all four subscales of the DLI, ‘practical knowledge’ was the strongest predictor (adjusted beta = 0.17) with an increase of 1 point in score, the FPR-EoL score increased by 0.63 (*p* < 0.01). However, ‘community support groups’ were negatively associated with FPR-EoL; with an increase of 1 point in score, the FPR-EoL score decreased by 0.23 (*p* < 0.01) (Table 4).

**Table 4:** Multiple linear regression models of predictors of FPR-EoL (behavior) (*n* = 2668)

		B	SE B	β	t	p-value	95% CI		VIF
Variables							Lower	Upper	
Constant		60.31	0.97		62.05	0.00	58.41	62.22	
Gender (ref.: Male)									
	Female	0.75	0.27	0.05	2.75	0.01	0.22	1.29	1.06
Age (year) (ref.: 18–34)									
	35–54	1.07	0.41	0.07	2.59	0.01	0.26	1.88	2.16
	55–74	0.87	1.35	0.01	0.64	0.52	-1.78	3.52	1.11
Highest Level of Education (ref.: High school or below)									
	College of higher	0.64	0.46	0.05	1.42	0.16	-0.25	1.54	4.13
Marital Status (ref.: Married/ cohabiting)									
	Single/ divorced/ separated/ widowed	-0.32	0.47	-0.02	-0.68	0.50	-1.25	0.60	3.56
Children (ref.: No children)									
	Yes	-1.27	0.50	-0.09	-2.56	0.01	-2.24	-0.30	3.55
Income (ref.: below city average)									
	Above city average	0.07	0.49	0.01	0.14	0.89	-0.90	1.04	4.78
Occupation (ref.: Medical (assistant) professional)									
	Student	-0.12	0.66	-0.01	-0.19	0.85	-1.41	1.17	8.86
	Other	0.74	0.38	0.05	1.94	0.05	-0.01	1.50	1.67
	Not employed	1.06	0.84	0.03	1.26	0.21	-0.58	2.71	1.42
Hospitalized within the past year (ref.: yes)									
	No	-0.02	0.57	0.00	-0.03	0.98	-1.13	1.10	1.03
Living with father (ref.: No)									
	Yes	-0.52	0.43	-0.04	-1.20	0.23	-1.36	0.33	3.99
Financial supporter of father (ref.: No)									
	Yes	-0.19	0.46	-0.02	-0.41	0.68	-1.10	0.72	4.29
Carer of father (ref.: No)									
	Yes	-0.30	0.46	-0.02	-0.64	0.52	-1.21	0.61	4.56
Father with disease (ref.: No)									
	Yes	0.39	0.29	0.03	1.33	0.18	-0.18	0.96	1.41
Father hospitalized within the past year (ref.: No)									
	Yes	-0.31	0.38	-0.02	-0.81	0.42	-1.04	0.43	1.14
Living with mother (ref.: No)									
	Yes	0.03	0.43	0.00	0.07	0.94	-0.81	0.88	3.97
Financial supporter of mother (ref.: No)									
	Yes	1.06	0.49	0.08	2.16	0.03	0.10	2.03	4.70
Carer of mother (ref.: No)									
	Yes	-0.73	0.49	-0.06	-1.50	0.13	-1.68	0.22	4.86
Mother with disease (ref.: No)									
	Yes	1.12	0.30	0.08	3.75	0.00	0.53	1.71	1.43
Mother hospitalized within the past year (ref.: No)									
	Yes	-0.24	0.41	-0.01	-0.58	0.56	-1.04	0.57	1.13
DLI scores									
	F1: Practical knowledge	0.63	0.09	0.17	7.23	0.00	0.46	0.80	1.62
	F2: Experiential knowledge	0.42	0.08	0.13	5.40	0.00	0.27	0.57	1.72
	F3: Factual knowledge	0.19	0.08	0.07	2.37	0.02	0.03	0.35	2.48
	F4: Community support groups	-0.23	0.07	-0.09	-3.16	0.00	-0.37	-0.09	2.33
*R* ^*2*^									0.14
*Adj. R* ^*2*^									0.13
*F*									16.71***
*df*									(25, 2642)

## Discussion and limitations

### The existence of discrepancies exists between filial attitudes and filial behaviors

The current study demonstrated a significant gap between the filial attitudes and filial behaviors of Chinese adult children toward their parents at the end stages of their lives. Children’s filial behaviors were stronger than their filial attitudes in three aspects, including ‘respect and comfort’, ‘acceptance of death’ and ‘spending final days’, indicating that they might be more willing to accept and to perform their filial duties when they are required to respect their parents [37]. An example item pertaining to this theme is ‘even if I do not agree to parents’ instructions about funeral arrangements, I would carry them out according to their wishes’. Another observation is that Chinese adult children tended to act less conservatively than indicated by their beliefs regarding filial piety in an EoL context; an example item in this context is ‘if doctors say that the treatments are no longer able to improve my parents’ condition, I would consider talking with my parents about stopping the treatments’. These findings indicated that if children prefer providing their dying parents comfort, they are more determined to execute it. From another perspective, children may suffer due to the struggle between their own filial attitudes and filial behaviors during the critical or the end stage of their parents’ lives. In addition, among all subscales of FPR-EoL, the behavior associated with disclosing bad news is significantly stronger than the attitudes of Chinese adult children. It could be explained that these adult children consciously agreed to disclose bad news to their parents, such as a terminal prognosis. However, when they truly put into action, they were reluctant to do so. Chinese adult children may tend to believe that a terminal prognosis could be devastating to their parents and that their parents could not withstand such a big shock [38]. Not only do they hesitate to inform their parents, they often object to others’ opinions including opinions from healthcare professionals, conveying them such bad news [38]. However, relevant studies in China have indicated that desiring others to tell the truth were higher in patients than in family members and medical staff [39, 40]. Patients’ willingness to be informed may be underestimated, and withholding the truth may prevent patients from knowing what they want to know during their final days of life. Chen’s study [41] even raised the concern that patients’ awareness of their terminal prognosis were more beneficial to bereaved family caregivers with regard to adapting to their grief. Difficulty in disclosing bad news poses the problem that dying patient may not be able to fulfill their last wishes while the bereaved family may experience more intense grief if their parents are unaware of their terminal prognosis, leading to reciprocal harmful consequences. Therefore, specific support for truth disclosure, for instance, professional training in disclosing bad news is suggested to be provided to Chinese families facing terminally ill situations. Trained family caregivers reported higher tendency to perform truth disclosure [38].

### Gender as a specific determinant of Chinese filiality in an EoL context

Gender stereotyping has traditionally influenced the dynamics and relationships between parents and children in the Chinese family system [42]. Notably, females (mother and daughter) are viewed as occupying an inferior position to that of males (father and son). Patriarchal parenting is a typical phenomenon in the child-parent relationships observed in some Chinese families [43]. The son of a family is even expected to bear higher filial obligations to care for his parents than a daughter does [44]. However, our study revealed that females exhibited a higher probability to perform filial behaviors than males in the context of parental EoL. This finding verified an observation previously reported [4], which indicated a reversal of the predominant role of daughters over sons in contemporary Chinese families. Females have undertaken the primary responsibility for providing care to their parents during the terminal stage [45, 46], and it is the concrete embodiment of filial behavior. This phenomenon can be comprehended and elucidated through various perspectives. Firstly, females typically shoulder a greater burden of household chores and caregiving duties within the family unit, and due to traditional gender roles that assign them nurturing responsibilities, they are more inclined to undertake the diverse caregiving tasks required in their parents’ final phase [47]. Secondly, females may also possess heightened emotional sensitivity and play a pivotal role in offering companionship to their parents during challenging times [48]. O’Neill [44] found that daughters exhibited stronger filial obligations due to the moderating effects of feelings of affection and gratitude, the binding of norms, and their own self-images in the context of emotional work. Moreover, particularly in Eastern cultures, filial piety is esteemed as a virtue and moral principle influenced by societal expectations and traditional beliefs, consequently, many females consciously or unconsciously embrace this significant responsibility and formed their filial perception [49, 50]. This situation represents either a great improvement in gender equality or a shift in filial obligations from the previous generation. This situation represents either a great improvement in gender equality or a shift in filial obligations from the previous generation.

Nevertheless, the findings of this study also indicated a higher probability among participants to exhibit filial representations if their father was dead rather than alive, while no such difference was noted in the case of mother’s death. This finding implies that Chinese adult children may usually not be able to exhibit their filial representations toward their living fathers. Owing to the authoritative role of fathers in Chinese families, children view their fathers as serious, respectful and, to a certain extent, relatively distant, leading to possible filial anxiety among children when communicating with their fathers [3, 19]. In Singapore, which is greatly influenced by filial norm, families’ adoption of an authoritarian filial piety approach has been found to be associated with poorer palliative care knowledge than family adoption of a reciprocal filial piety approach [17]. When faced with authoritative fathers, children tend to comply with their fathers’ directives, exhibit reluctance to voice their opinions, and more inclined owards performing certain “filial behaviors” in accordance with social norms, such as seeking ways to prolong their fathers’ life rather than addressing quality of life. Therefore, authoritarian father-child relationships could be an obstacle to the promotion of advanced care planning for the entire family. Lack of communication, palliative knowledge, and advanced care planning are likely to fail to guarantee quality EoL care. Moreover, a significant number of individuals in this study reported a higher probability to perform filial behaviors after their father has passed away. This is most likely due to the fact that such individuals have deeply reflected on father’s death and felt regretful of the ‘inadequate’ filial support that they provided to the dead father, which in turn may result in suffering from intense grief.

### Age maturity increases filial behaviors in an EoL context

The findings of this study indicated that age was relevant to filial piety representations in an EoL context. The older the Chinese adult children were, the more they perform filial piety behaviors to their parents. In traditional filial piety practices, adult children, especially sons, are expected to care for their older parents with regard to both daily living and periods of illness [42, 51]. In addition, senior parents occupy an authoritative position in the family hierarchy and should be fully respected [52]. On the other hand, as parents age, the remaining time that children can spend with their parents are viewed as less, which may also remind the children of their filial obligations toward the parents [4]. Meanwhile, as children age, they may become more mature or may have encountered the deaths of their peers’ parents, and thus become aware of their own greater filial obligation toward their parents. Older adult children tended to uphold the core values and traditional familial expectation than those who are younger in terms of providing direct care to parents [53]. They may also have retired and become more available to serve filial duties or they would like to be role-models to younger generation to continue the tradition of filial piety. Moreover, strong filial piety was identified as a protective factor that could reduce the negative impact of caregiving stress [47, 54], in spite of caregivers’ age and the care recipients’ dependency level [55]. The adult children may even experience a sense of gain, role reward or conformity to social morality after fulfillment of filial duties, which in turn buffers their caregiving burden [56, 57]. The beneficial effect of filial piety indicated the necessity of incorporating cultural values in supporting family, particularly in Eastern cultures. Provided that younger adult children were found to hesitate to engage in filial behaviors toward their dying parents in an EoL context, more guidance pertaining to filial representation should be provided to adult children, particularly to young adult children, when their parents are at the end stages of their lives. This approach may promote not only the quality of death of parents but also takes into account the adaptation of adult children to bereavement.

### Relationship with parents impacts filial behaviors in an EoL context

Intriguingly, our findings indicated that Chinese adult children who neither live with their parents, nor serve as carers and financial supporters of their parents exhibited significantly more filial behaviors in an EoL context. As the traditional filial duties of Chinese families require children to live with and care for their parents [58], children who do not live with their parents might consider EoL filial behaviors in an EoL context as way of compensating their parents during their last course of life [18]. Conversely, children who have played day-to-day caring and supporting roles for their parents might be less eager to fulfill more filial duties at the end stages of their parents’ lives. They may believe that they have been fulfilling their filial duties all along and that no specified reinforcement is needed during parents’ end-of-life. On the other hand, this finding verified the proverb ‘Even a filial child will eventually be fed up with taking care of a bedridden and chronically ill parent’. In these circumstances, the dying parents might be perceived as a stressor by the children who may become reluctant to take on more filial duties [59]. Such strong and pertain filial responsibilities may also adversely affect the mental health of caregivers when they are financially challenged [60]. Providing both practical and emotional support to carers in the EoL context appears to be important for achieving quality EoL care. Practical support such as assistive services, welfare incentives, respite care and emotional support such as relaxation programs and peer support group were recommended to provide to caregivers based on their socio-psychological background to alleviate their caregiving burden [47].

### Death literacy contributes to the filial behavior of adult children at the end of their parents’ lives

Our multiple linear regression analysis indicated that gender, experience in caregiving with the mother, and death literacy can contribute to the prediction of filial behavior at the parent’s EoL, which is consistent with findings suggesting that daughters exhibited more filial obligation than sons in the context of providing care to parents and that filial piety is a predictor of caregiving burden [15, 44, 54]. Thus, our findings revealed that death literacy can at least partially explain filial behavior in an EoL context, the entirety of such behavior still requires a great deal of further explanation. Among all the domains in the DLI, ‘practical knowledge’ was a relatively strong predictor of filial behavior in an EoL context. This factor reflected the individual’s perceived knowledge with regard to providing hands-on and talking support during death incidents [32], implying that people who exhibited confidence in their ability to personal support could demonstrate their filial behaviors toward their parents under EoL care. However, ‘community knowledge’ of DLI was a significant but mild predictor of EoL filial behavior in an EoL context. Their knowledge of community support groups were even negatively associated with filial behavior in an EoL context. This phenomenon implies that adequate EoL community support might be perceived by adult children as a way to relieve their filial burden [61, 62]. The availability of EoL community support might enable adult children to seek physical, psychological, mental, spiritual or other types of help for their dying parents, which is an elusive task and difficult to be achieved by the general public. In other words, adult children might even only be able to meet the needs of their dying parents by strengthening the commitment to EoL filial behaviors when lack of support from the community in EoL context. After decades of de-familialization experience of palliative care in the Nordic context, recent report emerged an argument for a need to strengthen the support for informal caring of family members who are important agency in EoL care [63]. Since, death literacy is a significant attribute to filial behaviors as represented in our findings, it highlights the need to enhance the clinical and social care networks of the death system in order to improve the death literacy of individuals and the provision of quality EoL healthcare, which in turn may help them to better perform filial duties, for instance arranging funeral and burial for dying parents [23, 29]. Therefore, improvement in death literacy may potentially have a positive influence on the filial behaviors of adult children with regard to their parents’ dying and death.

### Limitations

The study investigated a large sample, and as convenience sampling was adopted, nonrandomization and uneven distribution of the number of participants among five cities limited the generalizability of the results. Participants were relatively young, and their relative lack of experience of loss and grief may have resulted in lower EoL filial piety (behavior) in this study. The proportion of medical (assistant) professionals among the participants was slightly higher than of the proportion observed in the general population in the area. The day-to-day work of these professionals involves dealing with life and death as well as families and their grief. Accordingly, these professionals may have more pronounced feelings and perceptions regarding EoL filial piety as well as a higher level of death literacy. This fact may have led to potential bias in the results regarding to both filial piety in an EoL context and death literacy. Future studies should explore the filial piety of the general population of China in terms of EoL. Additional variables can be examined to identify their associations with and contributes to filiality in an EoL context.

## Conclusion

This study was one of the first investigations to explore the discrepancy between filial attitudes and filial behaviors in an EoL context and to examine the association between death literacy and filial behaviors pertaining to EoL. A significant gap between filial piety attitudes and behaviors was identified among Chinese adult Children at EoL of parents, notably with regard to disclosing bad news to their parents. Females, older Chinese adult children and children whose fathers were passed away, were found to exhibit a higher tendency of filial behaviors in EoL care for their parents. It is thus suggested to provide truth disclosure support to Chinese families as well as guidance to young adult children and to those who care for terminally ill fathers, which can promote reciprocal comfort for both children and parents in the final days of life under the filial norms. In addition, death literacy should be strengthened in the Chinese context through society-wide improvement and promotion of the death system, death services and grief support. Overall, filial behaviors in an EoL context can be improved among Chinese adult children, so as to potentially improve the quality of death of their parents.

## Data Availability

The datasets used and/or analyzed during the current study available from the corresponding author on reasonable request.
